# Anomalous Frontal Extra-Axial Midline Traversing Vein as the Potential Source of Subarachnoid Hemorrhage

**DOI:** 10.7759/cureus.25350

**Published:** 2022-05-26

**Authors:** Kristine Ravina, Mirhojjat Khorasanizadeh, Yu-Ming Chang, Christopher S Ogilvy, Ajith J Thomas

**Affiliations:** 1 Neurosurgery, Boston Medical Center, Boston, USA; 2 Neurosurgery, Beth Israel Deaconess Medical Center, Harvard Medical School, Boston, USA; 3 Radiology, Beth Israel Deaconess Medical Center, Harvard Medical School, Boston, USA; 4 Neurosurgery, Cooper Medical School of Rowan University, Camden, USA

**Keywords:** extra-axial developmental venous anomaly, anatomic variation, cerebral venous system, spontaneous subarachnoid hemorrhage, anomalous vein

## Abstract

Extra-axial developmental venous anomalies (DVAs) are important anatomic structures that contribute to supplemental venous drainage of intracranial contents into the extracranial veins. We present the case of a 35-year-old woman with a sudden-onset severe headache, nausea, and vomiting who was found to have an atraumatic subarachnoid hemorrhage of left frontal convexity. Workup revealed a large anomalous extra-axial vein originating in the right frontal area, traversing the left frontal region, penetrating the left frontal bone just above the supraorbital foramen with likely drainage into the left external jugular vein. This vein could not be classified as an emissary vein given the lack of direct communication with the superior sagittal sinus anterior portion, which was found to be hypoplastic. This case report adds to the literature a description of a previously unreported midline traversing frontal extra-axial vein directly draining frontal lobes with a potential implication in an atraumatic subarachnoid hemorrhage of frontal convexity.

## Introduction

Extra-axial developmental venous anomalies (DVAs) such as sinus pericranii, persistent embryonic sinuses, and emissary veins connect intracranial and extracranial veins [1]. These venous structures are relevant to neurosurgeons and neurointerventionalists, given their association with dural arteriovenous fistulas, infection transmission, and susceptibility to injury from trauma and surgical approaches. This report provides the first in literature description of a frontal extra-axial stand-alone venous anomaly not directly connected to an intracranial venous sinus.

## Case presentation

**Figure 1:**
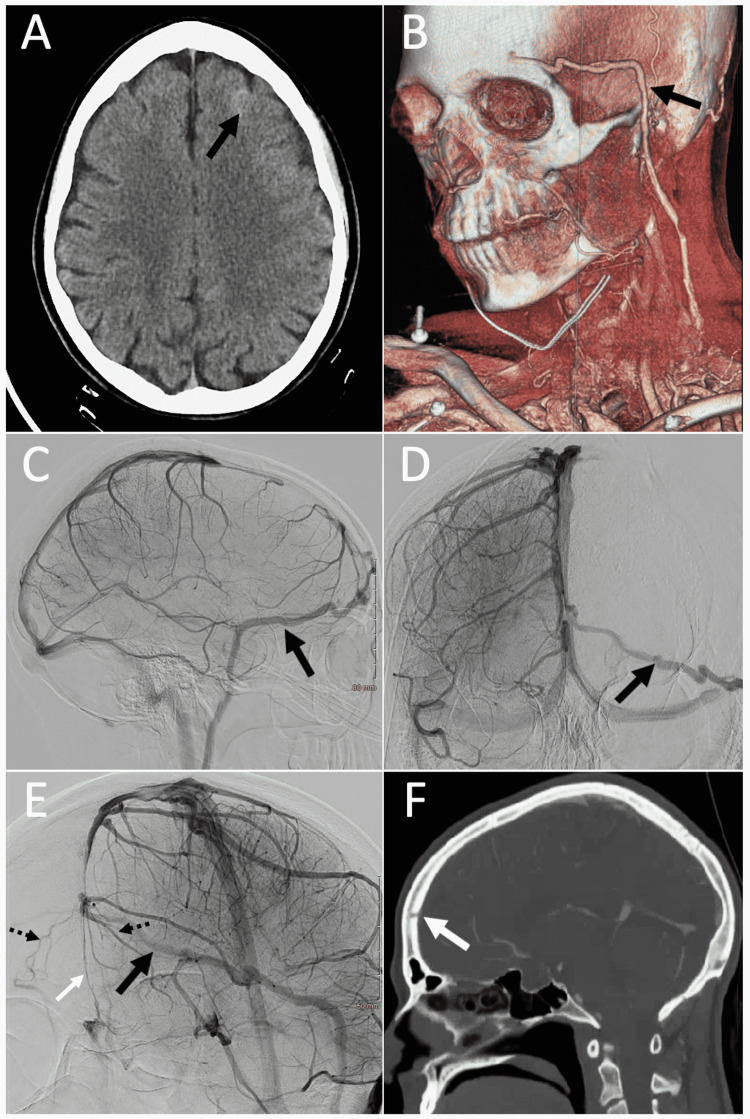
Imaging findings of the case presentation (A) Computed tomography (CT) scan showing left frontal subarachnoid hemorrhage (SAH) (black arrow). (B) 3-D imaging showing the exit of the anomalous vein through a foramen in the left frontal bone and then traveling superficially toward the neck. (C) Lateral projection of the right internal carotid artery angiogram showing the anomalous draining vein (black arrow). Note the hypoplastic anterior portion of the superior sagittal sinus. (D) Anterior-posterior projection of the right internal carotid artery angiogram showing the anomalous vein traversing from the right to the left side (black arrow). (E) Oblique projection of left internal carotid artery angiogram showing the anomalous vein (large black arrow) and its independence from the superior sagittal sinus notable more posteriorly. Additionally, the patient was noted to have a patent vein of foramen caecum (white arrow) and two smaller veins draining the bilateral orbital area into the venous confluence, then drained by the large anomalous frontal vein (dotted black arrows). (F) CT angiogram bone window demonstrating the abnormal osseous channel in the left frontal bone (white arrow).

A 35-year-old woman without any significant past medical history presented with sudden-onset “thunderclap” headaches while singing on a teleconference call, with subsequent nausea and vomiting, without a clear history of significant head trauma. The patient denied any history of headaches prior to this event. Upon presentation, the patient had a Glasgow Coma Scale score of 15 with no focal neurologic deficits. The initial CT angiogram revealed a left-sided superior frontal sulcus hyperdensity consistent with a small area of subarachnoid hemorrhage (SAH) (Hunt and Hess grade 1, modified Fisher grade 1; see Figure 1, Panel A). Magnetic resonance imaging (MRI) with angiogram was also performed, which demonstrated no evidence of an acute infarct, vasculitis, or reversible cerebral vasoconstriction syndrome (RCVS). Additionally, caput medusae venous configuration typically associated with cavernous malformations was not demonstrated [2].

Workup

A digital subtraction angiogram (DSA) was performed to further study the source of SAH. DSA revealed a large and tortuous anomalous vein originating in the right frontal area, traversing anteriorly and leftward into the left frontal region (Figure 1, Panels B-E). It then entered the skull through an osseous channel within the frontal bone (Figure 1, Panel F) and exited through a bony opening above the left supraorbital foramen in the left forehead. A more comprehensive evaluation of the vein showed an anomalous extra-axial stand-alone vein, which was responsible for a significant portion of frontal venous drainage as was demonstrated in the venous phase of right internal carotid artery DSA runs. The vein did not have a direct connection to the superior sagittal sinus (SSS) anterior part, which was found to be hypoplastic either as a result or the cause of the drainage provided by the anomalous vein. The exact terminus of the vein could not be identified with certainty, but it seemed to drain into the left external jugular vein at the level of the parotid gland. Additionally, the patient had a small, near-vertical anterior frontal vein that appeared to be the draining portion of nasal mucosa into the venous confluence drained by the large anomalous frontal vein in lieu of the SSS, most consistent with the vein of foramen caecum (Figure 1, Panel E) [3]. Small bilateral frontal veins connecting the orbital area with the anomalous venous confluence were also noted (Figure 1, Panel E).

Differential diagnosis

While this venous anomaly most closely resembled an emissary vein [4], there was no direct communication to the SSS and it appeared to be providing stand-alone drainage of a large portion of the frontal lobes. The possibility of this venous anomaly being a sinus pericranii was considered but eventually ruled out since sinus pericranii typically involves the formation of an extracranial varix or capillary network tightly adherent to the skull that communicates with an intracranial venous sinus via diploic veins [5], which was not visualized in this case. The patient had presented with hypertensive emergency and worsening headache that put RCVS or vasospasm among the differentials for the etiology of her presentation. Nevertheless, the patient did not have clear risk factors for RCVS, and the angiogram was negative for vasospasm.

Outcome and follow-up

The venous anomaly was thought to be the most likely etiology of the patient’s SAH. During hospitalization, she was treated with nimodipine, magnesium oxide, blood pressure control, and supportive measures. On follow-up, the headaches improved, but she still reported a mild residual ache behind her eyes with nocturnal worsening. However, there were no other neurologic symptoms. She also reported avoiding sleeping flat due to concerns about exerting pressure on the left-sided venous anomaly. The patient did not report any symptoms with Valsalva maneuver or straining. Four-week follow-up non-contrast head CT demonstrated resolution of the previously noted SAH.

## Discussion

Cerebral venous drainage occurs via the superficial and deep venous systems. The superficial bridging vein system provides venous drainage of the cortical regions, while the deep cerebral veins drain the deep gray and white matter [6]. The orbital surface of the frontal lobe is drained primarily into the SSS and to a lesser extent into the middle cerebral veins and the basal vein of Rosenthal via the anterior and posterior orbitofrontal, frontopolar, and olfactory veins [6-8]. As per a recent classification by Manjila et al., extra-axial developmental venous anomalies (eDVAs) represent a spectrum of venous anomalies including sinus pericranii, persistent embryonic sinuses, venous varices, venous aneurysmal malformations, and anomalous emissary veins, which are distinct from intra-axial DVAs typically associated with cavernous malformations [1,9]. Emissary veins are classically known to provide communication between the intracranial venous sinuses and extracranial veins passing through cranial apertures [1,4,7]. There have been only a few historical reports on emissary veins involving the anterior cranial fossa floor such as the vein of the foramen caecum, also found in the present case, connecting the nasal cavity to the SSS [7]. To our knowledge, this is a rare case report of a midline traversing stand-alone anomalous frontal vein not connected to an intracranial venous sinus. Instead of the right frontal lobe draining to the SSS, this anomalous vein provided independent drainage of the right frontal lobe extracranially through its own aperture in the left frontal bone traveling superficially on the temporalis muscle and then down toward the jugular vein (Figure 1, Panel B). Although this vein did not fulfill the criteria of an emissary vein since it did not have a clear connection with the SSS, it fits within the spectrum of eDVAs.

eDVAs such as the emissary veins have been implicated in several physiologic processes and neurosurgical pathologies including the normalization of intracranial pressure [4], brain cooling [10], development of dural arteriovenous fistulas [11], pseudotumor cerebri [12], and intracranial spread of infections, given their valveless nature [4]. Sinus pericranii is another type of eDVA that is formed by venous tributaries typically converging into a diploic lake or dural venous sinus that communicates to a subcutaneous venous varix [1,13]. An important consideration is the path of travel of the vein since its superficial nature can make it susceptible to injury as it is not protected by muscle or bone along its course. For example, blunt force trauma or significant external pressure to the left side of the neck, left temporal region, or forehead, in this case, can potentially cause venous injury or flow disturbances leading to significant hemorrhage, venous thrombosis, or cerebral venous infarct similar to jugular vein injury from trauma to the neck or improper surgical positioning [14,15].

Surgical approaches should be carefully planned, and pre-operative vascular imaging was reviewed taking into consideration the location and anatomic relationships of eDVAs to the bony anatomy and soft tissue structures, given the risk of complications in case of iatrogenic obliteration or manipulation [14,16,17]. Given these relevant associations, understanding of eDVAs and their variations is important when considering open or endovascular neurosurgical interventions.

## Conclusions

The frontal extra-axial stand-alone midline traversing eDVA described in this report provides venous drainage of the frontal brain parenchyma directly into the external jugular vein and, to our knowledge, has not been previously reported. This case report contributes to the limited literature on frontal cerebral venous anomalies. Knowledge of anomalous veins and their anatomic associations is relevant for neurosurgeons and neurointerventionalists, given their potential role in brain physiology, pathology, and surgical complications.
